# Supplementary figures and images for: Inhibition of TOR in Chlamydomonas reinhardtii Leads to Rapid Cysteine Oxidation Reflecting Sustained Physiological Changes
Source: Cells. 2019 Sep 28;8(10):1171. doi: 10.3390/cells8101171 (PMC6829209; doi:10.3390/cells8101171)

a.

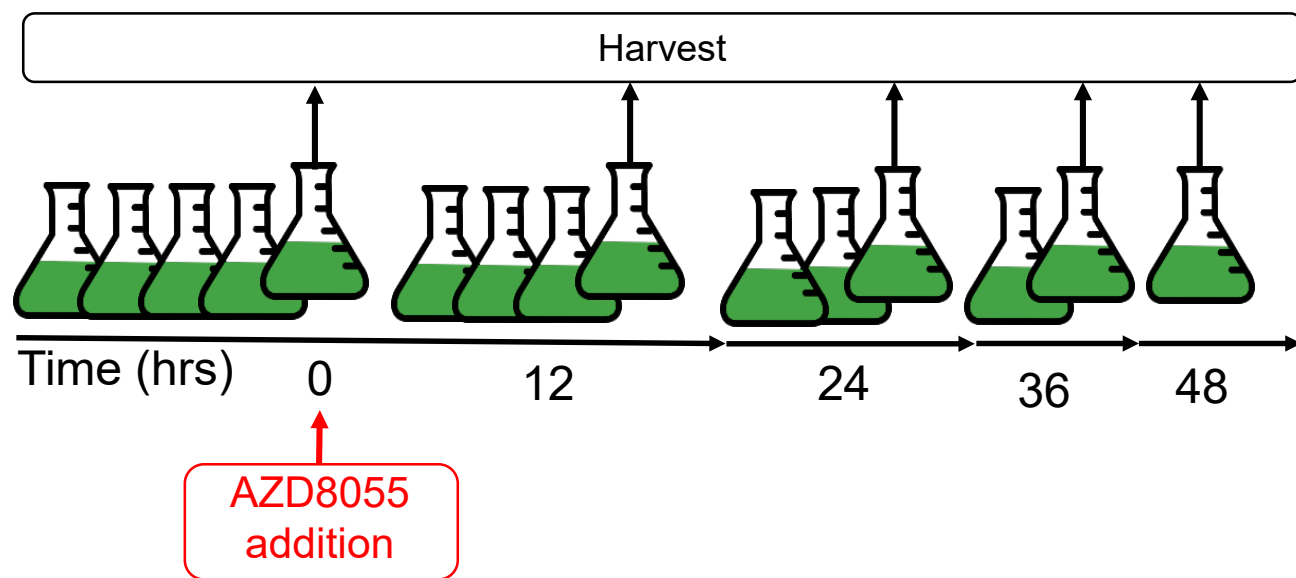

b.

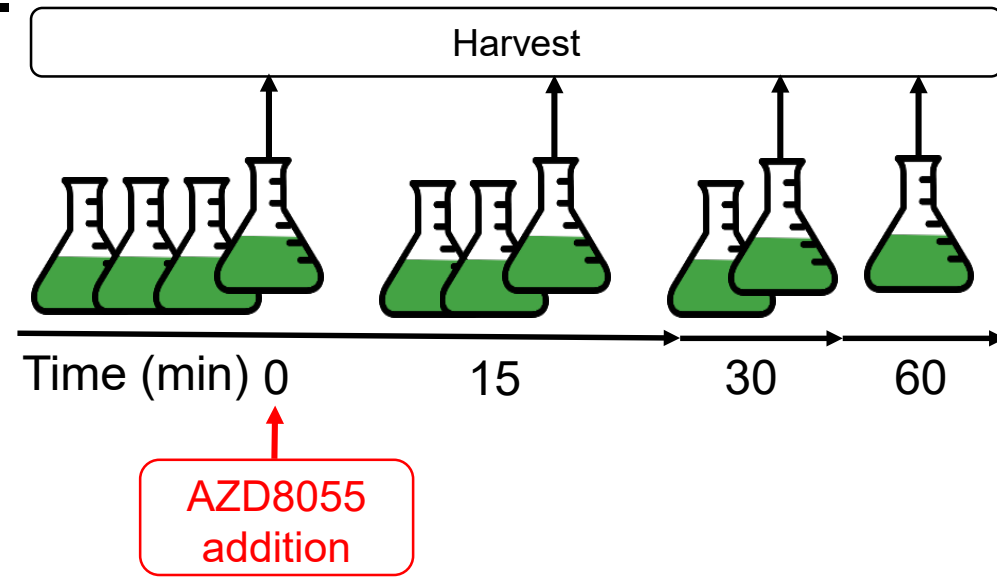

Supplement: Supplementary file 1 [file cells-08-01171-s001.zip › Cells_MMFord_SupplementaryFiles/Cells_MMFord_FigS1.pdf]

# Cell number

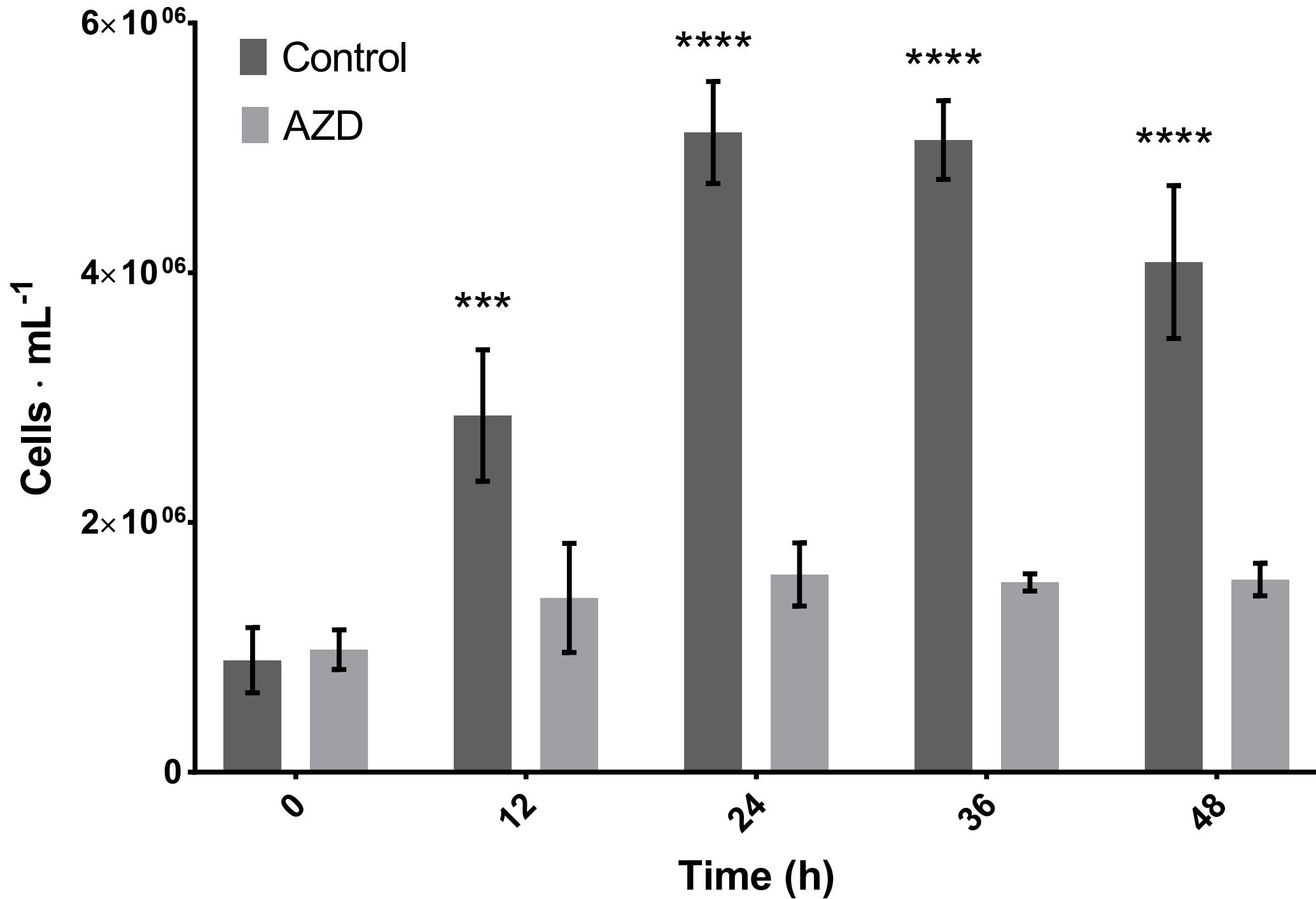

Supplement: Supplementary file 1 [file cells-08-01171-s001.zip › Cells_MMFord_SupplementaryFiles/Cells_MMFord_FigS2.pdf]

**B<sub>0</sub>**

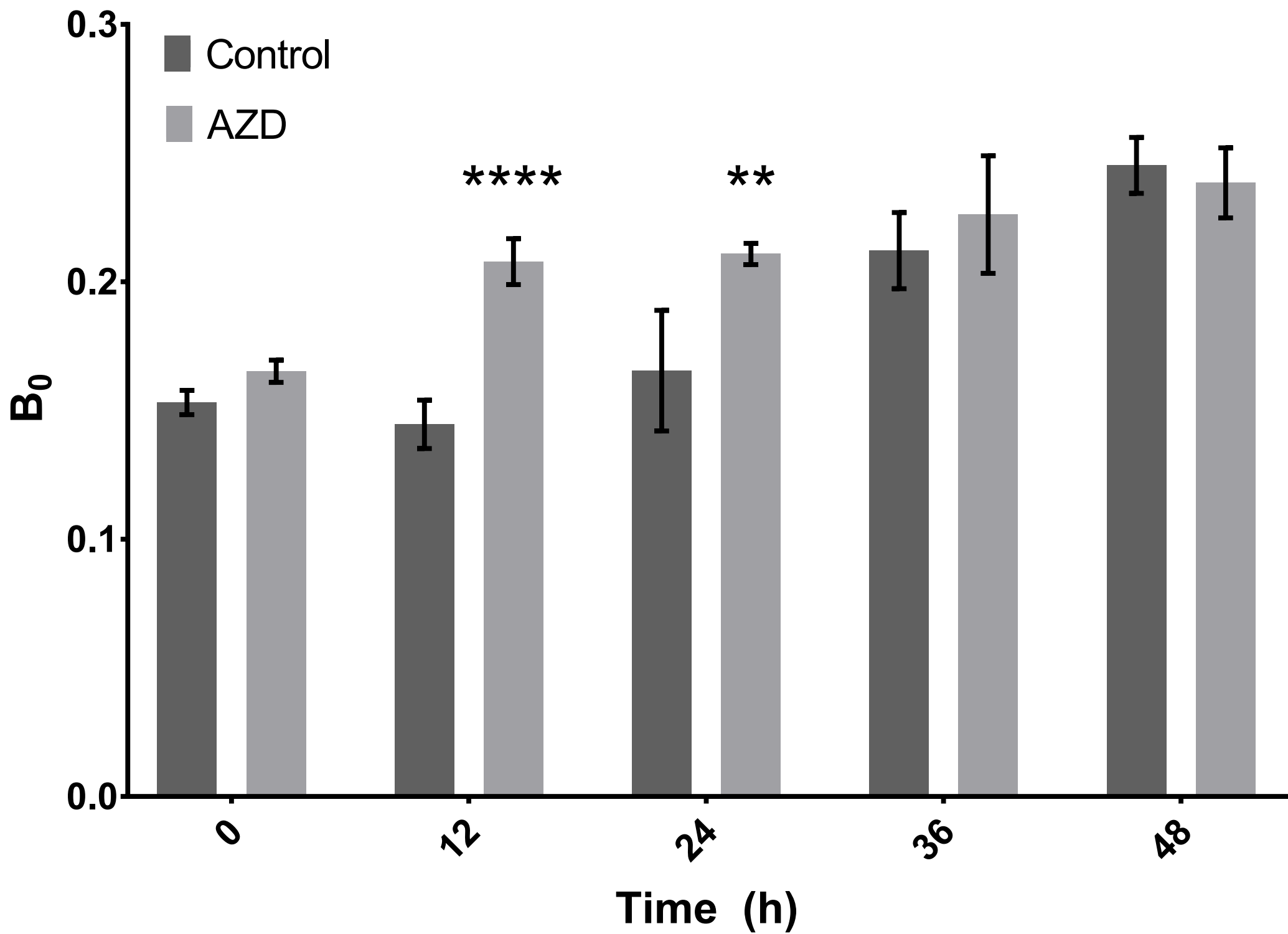

Supplement: Supplementary file 1 [file cells-08-01171-s001.zip › Cells_MMFord_SupplementaryFiles/Cells_MMFord_FigS3.pdf]

a.

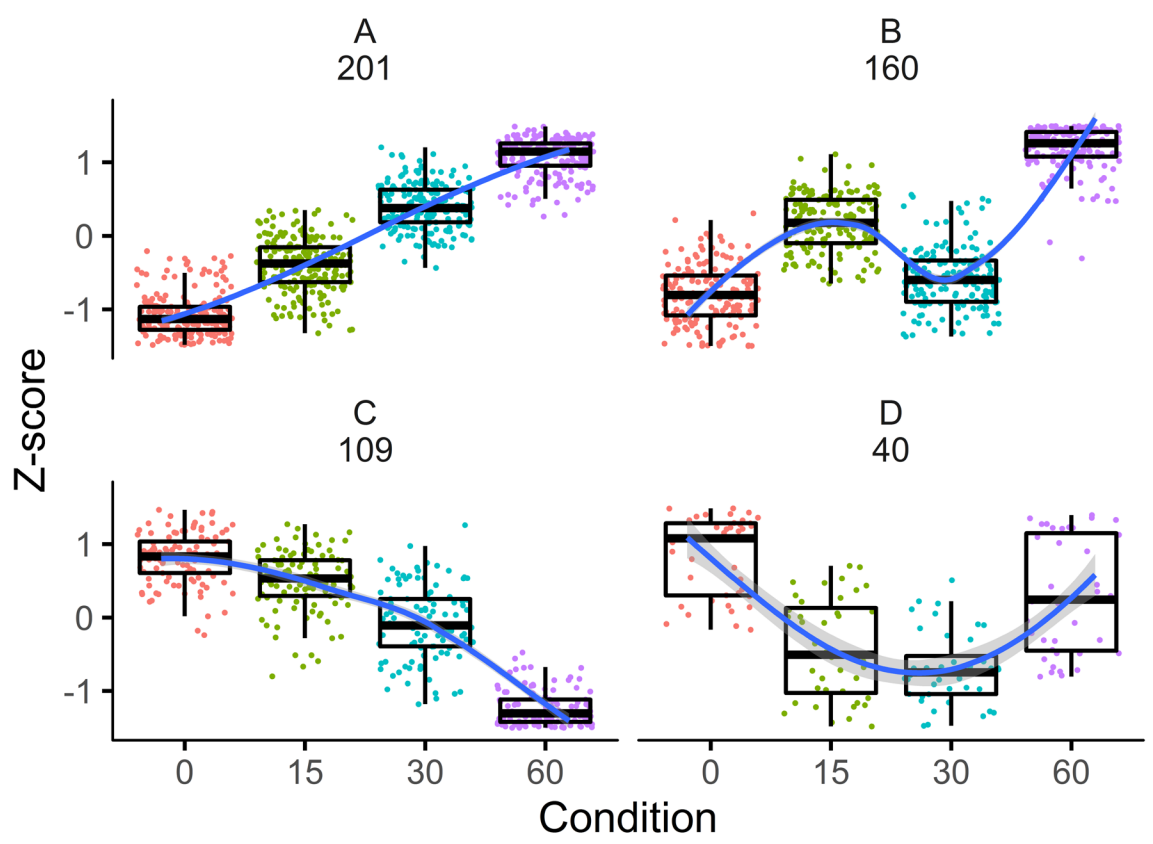

b.

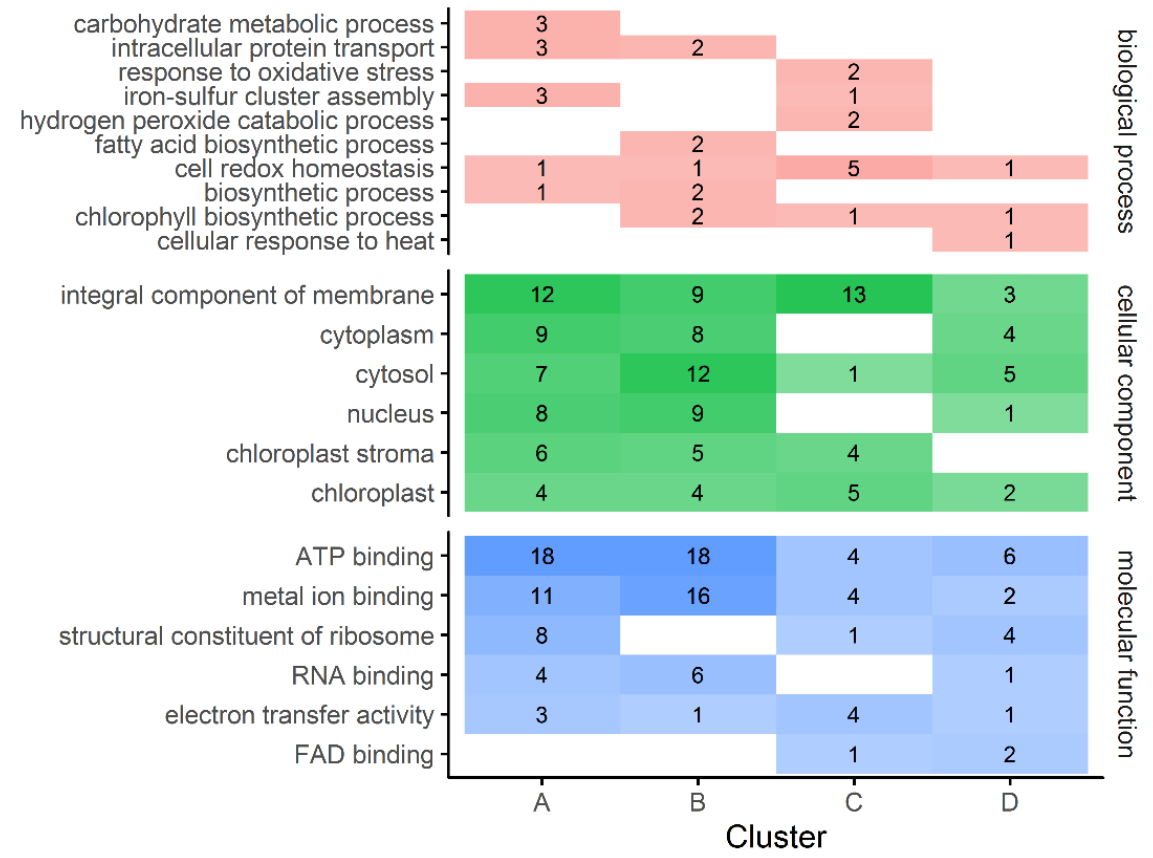

Supplement: Supplementary file 1 [file cells-08-01171-s001.zip › Cells_MMFord_SupplementaryFiles/Cells_MMFord_FigS4.pdf]
